# Munda languages are father tongues, but Japanese and Korean are not

**DOI:** 10.1017/ehs.2020.14

**Published:** 2020-05-29

**Authors:** Gyaneshwer Chaubey, George van Driem

**Affiliations:** 1 Department of Zoology, Benaras Hindu University, Varanasi, Uttar Pradesh 221005, India; 2 Linguistics Institute, University of Bern, Länggassstrasse 49, CH 3012 Bern, Switzerland

**Keywords:** Austroasiatic, Japanese, Munda, population genetics, ethnolinguistic prehistory

## Abstract

Over two decades ago, it was observed that the linguistic affinity of the language spoken by a particular population tended to correlate with the predominant paternal, i.e. Y-chromosomal, lineage found in that population. Such correlations were found to be ubiquitous but not universal, and the striking exceptions to such conspicuous patterns of correlation between linguistic and genetic phylogeography elicit particular interest and beg for clarification. Within the Austroasiatic language family, the Munda languages are a clear-cut case of father tongues, whereas Japanese and Korean are manifestly not. In this study, the cases of Munda and Japanese are juxtaposed. A holistic understanding of these contrasting cases of ethnolinguistic prehistory with respect to the father tongue correlation will first necessitate a brief exposition of the phylogeography of the Y chromosomal lineage O. Then triangulation discloses some contours and particulars of both long lost episodes of ethnolinguistic prehistory.

## Graphical abstract

**Figure:**
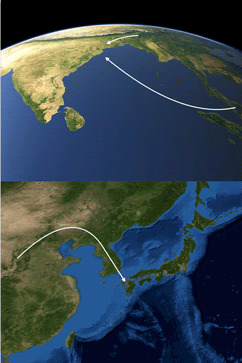

**Media summary:** The origins of the Japanese and the origins of the Munda peoples of India provide contrasting cases of ethnolinguistic prehistory.

## The uneasy relationship between language and the Y chromosome

The observation that the linguistic affinity of the language spoken by a particular population tends often to correlate with the predominant Y-chromosomal lineage found in that population was first pointed out by a Swiss–Italian team of geneticists (Poloni *et al.* 1997, 2000). As Lendering (2010: 252) observed in his history of Alexander the Great, ‘As so often happened in the wars of antiquity, the widows married the murderers of their spouses’. Historical accounts record that the campaigns led by Genghis Khan, Tamerlane and other conquerors availed themselves of the same tactic, and Kivisild (2014) has wryly qualified warfare as a Y chromosome-linked pathology.

Although the motif of male genocide has repeated itself throughout history, conquering incursive groups might very well have been more clement in many particular instances, yet may nonetheless have benefitted from preferential access to the womenfolk and a more prolific siring of progeny by dint of élite dominance. As we have noted before, the preponderance of such correlations allows us to deduce that a mother teaching her children their father's tongue must have been a prevalent and recurrent pattern in linguistic prehistory. As a consequence of this social and demographic mechanism, paternally inherited polymorphisms often serve as tracers for linguistic dispersals in the past (van Driem 2007), and a particular Asian subset of such patterns of correlation forms the topic of the present paper.

Furthermore, the shallower time depth of the linguistically reconstructible past and the putative age of recognised language families match the time depth attributed to the most recent common ancestor of many geographically widespread paternal lineages (Zerjal *et al.* 2003; Balaresque *et al.* 2015). However, a few other forces are hypothesised to influence this topology equally (Karmin *et al*. 2015). In sharp contrast with our mitochondrial past and also in comparison with the rest of the genome, Y chromosomal phylogeography is relatively recent, having undergone a global bottleneck towards the end of the last ice age, when a subset of the then existing paternal clades began eradicating and out-competing other clades (Karmin *et al.* 2015; Silva et al. 2017). Effective palaeolithic founder populations of major modern paternal subclades were small. In terms of Y-chromosomal lineages, entire new populations arose from small surviving subsets that had passed through such bottlenecks. Similarly, many of today's language families and linguistic phyla appear to be the result of prehistoric bottlenecks.

When languages and genes happen to exhibit a correlation, such a relationship ought not to be confused with identity, and a chromosomal marker should not be simplistically equated with populations speaking languages of a particular linguistic phylum. Rather, markers on the Y chromosome serve as proxies or tracers for the movements of paternal ancestors. Although ubiquitous, the father tongue correlation is not universal. Exceptions to the father tongue correlation, such as Hungary and Baltistan (van Driem 2007), are not unique cases. Rather, the meticulous study of correlations of genetic polymorphisms with the geographical distribution of language families and their constituent subclades as well as the discrepancies between genetic and linguistic phylogeography, nowadays enriched with the information rendered available through whole genome studies and ancient DNA findings, are providing us with an increasingly differentiated view of the past with ever greater detail.

## The East Asian linguistic phylum

**Figure 1.**
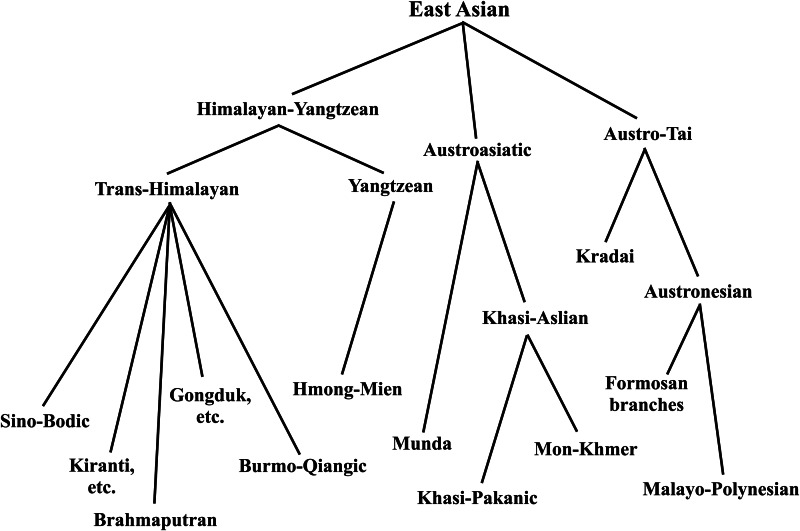
The enhanced 2012 Benares recension of Starosta's East Asian linguistic phylum (Starosta 2005; van Driem 2014).

The East Asian linguistic phylum was proposed by Starosta in Périgueux in 2001, one year before his death. His thinking was published posthumously, shown in Figure 1 (Starosta 2005; van Driem 2005). In proposing to unite the Kradai, Austronesian, Trans-Himalayan, Hmong-Mien and Austroasiatic language families into a single East Asian linguistic phylum, Starosta had numerous precursors. Conrady (1916, 1922) and Wulff (1934, 1942) proposed a linguistic phylum consisting of Austroasiatic, Austronesian, Kradai and Trans-Himalayan, whilst Benedict (1942), Blust (1996) and Peiros (1998) proposed an Austric superfamily comprising Austroasiatic, Austronesian, Kradai and possibly Hmong-Mien. The more modest proposal to unite just two of these five East Asian language families, viz. Kradai and Austronesian, was first advanced by Schlegel (1901, 1902) and then seconded by Benedict (1942). However, the first sound historical comparative evidence for the Austro-Tai family was adduced by Ostapirat (2005, 2013).

For his grander East Asian linguistic phylum, Starosta adduced the putative shared morphological vestiges of an agentive prefix *<m->, patient suffix *<-n>, instrumental prefix <s-> and perfective prefix *<n->. A discussion of the merits of this evidence strikes us as being of little utility, since we consider the antiquity of the proposed linguistic phylum to lie at the ‘linguistic event horizon’ or maximal time depth reconstructible through methodologically sound historical linguistic comparison. Beyond this epistemological boundary hypotheses regarding long-distance linguistic relationship are reduced to sheer speculation. Rather, Starosta (2005: 194) modestly proposed that the ‘potential utility’ of his hypothesis lay ‘in helping to focus scholars’ efforts on particular specific questions, resulting in the replacement of parts of this hypothesis with better supported arguments’. Following Ostapirat, Starosta's East Asian linguistic phylum may be construed to comprise four recognised language families: Austro-Tai, Trans-Himalayan, Hmong-Mien and Austroasiatic.

## The Story of O

Populations today speaking languages of the Trans-Himalayan, Hmong-Mien, Austroasiatic and Austro-Tai language families happen to be characterised by a preponderance of the paternal lineage O. In fact, each of the four language families are characterised by a particular subclade of O, suggesting both a paternal spread of these language families and a time depth for the putative East Asian language family coeval with the antiquity of the paternal haplogroup O itself. There is good reason to believe that the geographical locus of the ancestral haplogroup NO (M214) lay in the eastern Himalayan region, where the two paternal lineages N and O split up (Karmin *et al.* 2015; Ilumäe *et al.* 2016; McColl *et al.* 2018).

We previously identified the clade N (M231) with the paternal spread of Fortescue's Uralo-Siberian linguistic phylum (van Driem 2014). The bearers of haplogroup N set out for East Asia just after the Last Glacial Maximum, braving ice and tundra, and in a grand counterclockwise sweep, gradually migrated across northern Eurasia as far west as Lappland (Rootsi *et al.* 2007; Derenko *et al.* 2007; Mirabal *et al.* 2009; Ilumäe *et al.* 2016), whilst the ancestral form *N may originally have been situated in northern Burma, Yúnnán and Sìchuān. Recent genetic studies have provided evidence that corroborates our earlier linguistic conjecture that the westward spread of this linguistic phylum across northern Eurasia must have involved the linguistic assimilation of earlier populations already residing in Siberia (Tambets *et al.* 2018; Lamnidis *et al.* 2018; Günther *et al.* 2018; Saag *et al.* 2019).

**Figure 2.**
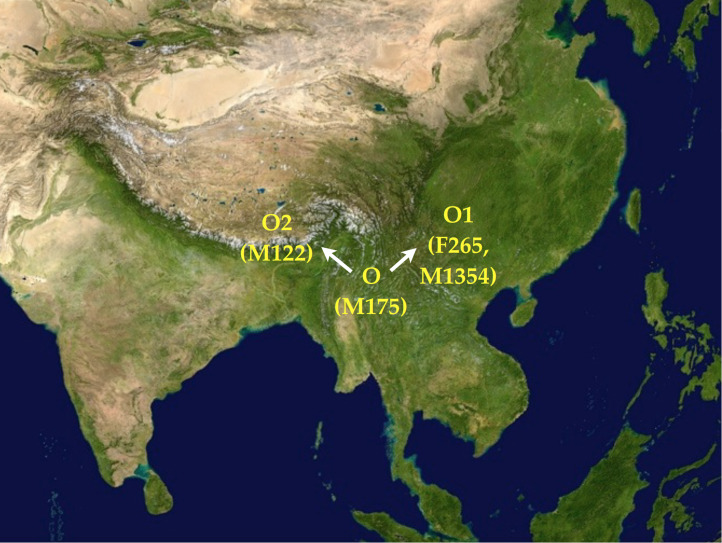
After the Last Glacial Maximum, the Y chromosomal haplogroup O (M175) split into the subclades O1 (F265, M1354) and O2 (M122).

The dissemination of Y chromosomal haplogroup O (M175) throughout East Asia from the eastern Himalayan region, as temperature and humidity increased after the Last Glacial Maximum, has been recounted in detail before (van Driem 2014) and will be recapitulated here in updated form. The entirely non-random correlation of subclades of this particular paternal lineage with populations speaking languages of the Trans-Himalayan, Austro-Tai, Hmong-Mien and Austroasiatic families enables us to infer the following sequence of events. Before the end of the Last Glacial Maximum, the paternal lineage O (M175) split into the subclades O2 (M122) and O1 (F265, M1354), as shown in Figure 2. The two subclades can be putatively assigned to two geographical loci, with the haplogroup O1 (F265, M1354) moving eastward into East Asia south of the Yangtze, whilst bearers of the O2 (M122) haplogroup settled in the eastern Himalayan region. Subsequently, over the course of time, as temperature and humidity increased after the last glacial maximum, haplogroup O split further into the paternal lineages that serve as tracers for the spread of Trans-Himalayan, Hmong-Mien, Austroasiatic and Austro-Tai.

The O1 (F265, M1354) lineage south of the Yangtze split into the subclades O1b (M268) and O1a (M119), with the latter moving eastward to the Fújiàn hill tracts and across the strait to settle on Formosa, which so became the *Urheimat* of the Austronesians. The founding dispersal of the Austro-Tai language family can be traced through a correlation of the current geographical range of Austro-Tai languages with the chronology and spread of the molecular proxies defining the paternal haplogroups O1b (M268) and O1a (M119). Just as has long been thought (Skeat and Blagden 1906), Asian negritos reflect an older layer of peopling (Chaubey and Endicott 2013; Jinam *et al.* 2017), and the peoples of insular Southeast Asia represent a patchwork quilt resulting from layers of peopling, with the Austronesian expansion from Formosa overlaying earlier strata of peopling (Mörseburg *et al.* 2016).

Recently, an exploration of Southeast Asian prehistory (Lipson *et al.* 2018) was quickly superseded by a more detailed study (McColl *et al.* 2018). The findings of both studies are commensurate with the model of a linguistic dispersal emanating from Formosa through insular Southeast Asia to the Southeast Asian mainland, Madagascar and Oceania, for which the geographical spread of the paternal lineage O1a (M119) serves as a molecular tracer. In this context, the Papuan ancestry in the Southwest Pacific appears to reflect a layer of peopling more recent than the initial population. The bearers of the Papuan ancestral component arrived either in belated emulation of the original Austronesian seafarers or as part of later waves of mixed migration (Skoglund *et al.* 2016).

**Figure 3.**
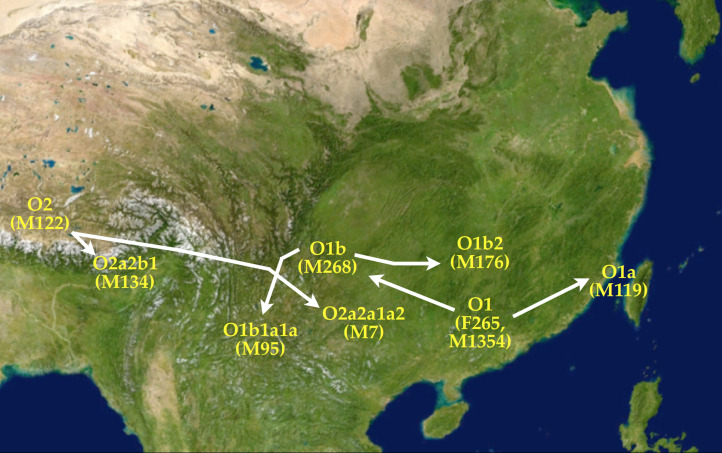
Paternal lineages branching into new subclades. Each event involved a linguistic bottleneck leading to language families that today are reconstructible as distinct linguistic phyla. The O1 (F265, M1354) lineage gave rise to the O1a (M119) subclade, which moved eastward to the Fújiàn hill tracts and across the strait to Formosa, which so became the *Urheimat* of the Austronesians. Bearers of the O1b (M268) paternal lineage domesticated Asian rice. Bearers of O2a2a1a2 (M7) became the Proto-Hmong-Mien. In the Eastern Himalaya, the bearers of haplogroup O2a2b1 (M134) expanded and became the Trans-Himalayans. Haplogroup O1b1a1a (M95) is the Proto-Austroasiatic paternal lineage. The para-Austroasiatic fraternal clade O1b2 (M176) spread eastward, sowing seed along the way.

Subsequent to the split-up of the paternal lineage O1 (F265, M1354) into the subclades O1b (M268) and O1a (M119), the paternal subclade O1b (M268) gave rise to the filial subclades O1b2 (M176) and O1b1a1a (M95). The bearers of haplogroup O1b1a1a (M95) became the progenitors of the Austroasiatics (van Driem 2007). The Austroasiatics spread throughout the Salween drainage and thence to southern Yúnnán, northern Thailand and western Laos. In time, the Austroasiatics would spread as far as the Mekong delta, the Malay peninsula and the Nicobars, and their paternal lineage would also spread deep into insular Southeast Asia. However, the prominent paternal lineage O1b2 (M176), previously referred to as ‘para-Austroasiatic’, does not appear to be correlated with any extant linguistic group today (Figure 3).

The spread of haplogroup O1 (F265, M1354) reflects the paternal founding dispersals of both Austro-Tai and Austroasiatic as well as the geographical spread of a para-Austroasiatic paternal subclade that evidently left no modern linguistic descendants. Our data from the Himalayan region and the data from populations elsewhere in Asia indicate that the geographical range and the chronology of spread of haplogroup O2a2b1 (M134) trace the founding dispersal of the Trans-Himalayan language family, whereas the paternal lineage O2a2a1a2 (M7) serves as a molecular proxy for the founding and spread of Hmong-Mien.

About 12,000 years ago, at the dawn of the Holocene, in the southeastern Himalayas and eastern slopes of the Tibetan Plateau, haplogroup O2 (M122) gave rise to the ancestral Trans-Himalayan paternal lineage O2a2b1 (M134) and the ‘Yangtzean’ or Hmong-Mien paternal lineage O2a2a1a2 (M7). The bearers of the polymorphism O2a2b1 (M134) at first remained in the Eastern Himalaya, which today continues to represent the centre of phylogenetic and linguistic diversity of the Trans-Himalayan language family based on the geographical distribution of primary linguistic subgroups. Subsequently, after bearers of the O2a2a1a2 (M7) lineage migrated eastward to settle in areas south of the Yangtze, they were followed by early Trans-Himalayan language communities that spread from northeastern India into southeastern Tibet and northern Burma.

## Austroasiatic seafarers set sail for the Subcontinent

The unity of Austroasiatic as a linguistic family has always been in evidence ever since Mason (1854), even though this linguistic finding flew in the face of the pronounced phenotypical disparity readily observed between Austroasiatic language communities. Schmidt (1906) proposed the idea of an Austroasiatic ‘race’, but Blagden (1909) rejected both Schmidt's Austric theory and assailed his notion of an Austroasiatic ‘racial stock’, stressing instead the demonstrable linguistic unity of the Austroasiatic language family.

Whether or not there is a thin strain of common blood running through these very diverse races is a point that does not and cannot affect the classification of their languages. Personally I rather regret that the attempt has been made to establish even a qualified racial unity such as this amongst populations which differ physically amongst themselves as much as chalk does from cheese. Not only is it in my judgement premature inasmuch as the data available are quite inadequate to support the conclusion, but it is liable to do harm by casting doubt on the validity of the purely linguistic inferences, where the evidence is far more perfect. (Blagden 1909: 172)

Schmidt's adversary in Vienna, Robert von-Heine-Geldern (1920), likewise dismissed his Austroasiatic ‘race’ as untenable, and hastened to point out that the phenotypical diversity between populations speaking Austroasiatic languages contrasted with the conspicuous lack of quantifiable phenotypical differences that could be observed ‘zwischen den Austroasiaten, Tibeto-Birmanen und Siamo-Chinesen Birmas, Assams und der Chittagong Hill Tracts’. The history of science has now partly vindicated both Schmidt and his rivals, for, whilst there is no such thing as ‘race’, let alone any such thing as an Austroasiatic ‘race’, the once mooted ‘thin strain of common blood running through these very diverse races’ has actually been found in the shape of molecular genetic evidence indicating that, secondarily, male Austroasiatics introduced both their language and their paternal lineage, O1b1a1a (M95), to the indigenous peoples of the Choṭā Nāgpur.

We first showed (Chaubey *et al.* 2011) that the Munda branch of Austroasiatic had arisen as the result of a sexually biased linguistic intrusion into the Indian subcontinent from Southeast Asia, and our findings have been corroborated by subsequent studies (Arunkumar *et al.* 2015; Metspalu *et al.* 2018; Tätte *et al.* 2019). As a consequence of the comparatively younger date and the highly pronounced gender asymmetry of this linguistic intrusion, it appears that the deepest division within the Khasi-Aslian trunk of Austroasiatic, i.e. the split between Khasi-Pakanic and Mon-Khmer, would be indicative of the geographical location of the Austroasiatic homeland, rather than the split between Munda and Khasi-Aslian. Therefore, the point of dispersal for Khasi-Aslian would appear to have lain in the area between South Asia proper and mainland Southeast Asia proper.

Rau and Sidwell (2019) have advanced the daring Munda maritime hypothesis, proposing that the male linguistic ancestors of the Munda migrated by sea to the Orissan coast from the Tenasserim or the Isthmus of Kra or even from the South China Sea littoral of mainland Southeast Asia and thence through the Straits of Malacca to the Indian subcontinent. Their hypothesis is inspired by Chaubey *et al.* (2011), but their linguistic data are gossamer and limited to a few lexemes pertaining to rice, millet agriculture and livestock. A new genetic study (Tätte *et al.* 2019) has lent support to this bold hypothesis in that the Munda show the highest sharing of identity-by-descent segments with Austroasiatic tribal groups on the Malay peninsula. The presence at Orissan coastal sites of knobbed and rouletted ware, which formed part of the maritime trade between South and Southeast Asia, is likewise suggestive (Tripati *et al.* 2019). Based on these findings, we would suggest that it might prove fruitful to compare Munda more particularly with Proto-Nico-Monic.

**Figure 4.**
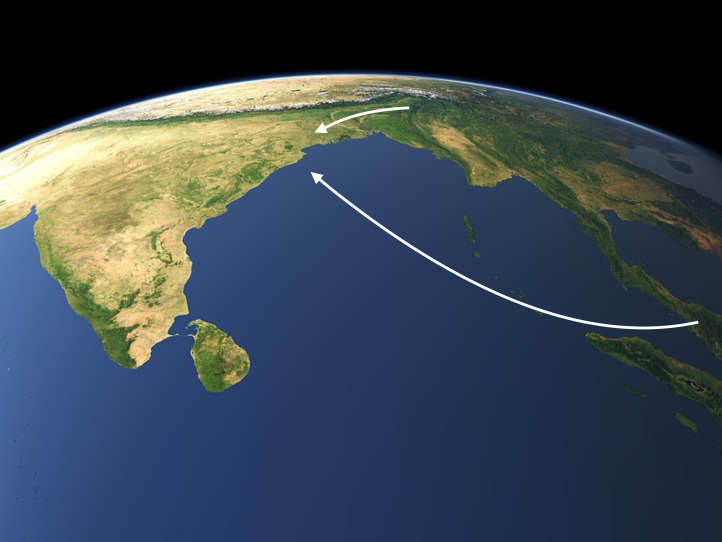
Two versions are depicted of the male-biased migration which introduced Austroasiatic language and four O1b1a1a1 (M95) paternal lineages to indigenous populations of the Choṭā Nāgpur, the overland trek from the Meghālaya originally proposed by Chaubey *et al.* (2011) and the Munda maritime migration proposed by Rau and Sidwell (2019).

However, a new paper (Singh *et al.* forthcoming), which specifically examines Austroasiatic populations of the Subcontinent, has identified three founding paternal lineages common to both Khasi and Munda speaking populations. Yet the number of Khasi individuals analysed was small, and more Khasi individuals will need to be analysed. Figure 4 therefore depicts two versions of the male-biased Austroasiatic linguistic intrusion that established Munda languages in India, the northern trajectory originally proposed by Chaubey *et al.* (2011) and the new southern Munda maritime exodus, with the Nicobar archipelago shown lying squarely in the path of the seafarers’ course. Both arrows of migration depict Munda languages as father tongues, whether brought to India by men from the Meghālaya or spawned by Austroasiatic seamen.

The population genetics of Asian rice has suggested three distinct domestication events involving the *ahu*, *indica* and *japonica* rice cultivars (Londo *et al.* 2006; McNally *et al.* 2009; Civáň *et al*. 2015). Based on linguistic palaeontological evidence (van Driem 2017), the linguistic ancestors of the Austroasiatics and the Hmong-Mien or ‘Yangtzeans’ were identified as the people behind two of these three domestications. The hypothesis was argued that the ‘para-Austroasiatic’ bearers of Y-chromosomal haplogroup O1b2 (M176), might have been the actors behind a third domestication. A large rice population genetic (Wang *et al.* 2013) has meanwhile lent additional support to our reconstruction involving several distinct rice domestications. Bearers of the para-Austroasiatic paternal lineage advanced as far as the Korean peninsula and became a major contributor to the Japanese genome, representing the probable paternal lineage of the Yayoi people, who introduced rice agriculture to the Japanese archipelago, as early as the second millennium BC, during the final phase of the Jōmon period.

## The story of O continues

Intimate interaction between ancient Austroasiatics and the ancestral Hmong-Mien not only involved the sharing of knowledge about rice agriculture, but also left a genetic trace in the high frequency of haplogroup O1b1a1a (M95) in modern Hmong-Mien populations and of haplogroup O2a2a1a2 (M7) in Austroasiatic populations. Further support for our 2017 rice domestication model has been provided by the most recent study of mainland Southeast Asia, where early Mesolithic male and female demographic expansions are reported to have taken place 10,000 years ago, whereas subsequent population prehistory shows contrasting male and female genetic histories. A major male-specific expansion involving Y-chromosomal haplogroup O1b1a1a (M95) took place in the Neolithic period between 5000 and 4000 years ago, reflected in modern Austroasiatic language communities. A second major male-specific expansion transpired during the Bronze and Iron Age between 2500 and 2000 years ago, involving Y-chromosomal haplogroup O2a (M324), seen in modern Kradai language communities in this region (Kutanan *et al.* 2018a, b, 2019).

Meanwhile, bearers of Y chromosomal haplogroup O2a2b1 (M134) in the eastern Himalayan region expanded eastward throughout Sìchuān and Yúnnán, north and northwest across the Tibetan plateau as well as westward across the Himalayas and southward into the Indo-Burmese borderlands. On the Brahmaputra plain, Trans-Himalayans encountered the Austroasiatics, who had preceded them. The relative frequencies of the Y chromosomal haplogroup O1b1a1a (M95) in Bodo-Koch language communities (Sahoo *et al.* 2006; Reddy *et al.* 2007) suggest that these Trans-Himalayan populations of the Indian subcontinent included and assimilated male Austroasiatic speakers in the past.

Finally, the Trans-Himalayan paternal lineage O2a2b1 (M134) spread northeast to the North China plain. The complex history of Sinitic populations featured successive constellations of dynastic empires governed from geographically ever shifting capitals, whereby subjugated and neighbouring populations as well as immigrants were absorbed. Consequently, Hàn Chinese populations comprise an amalgam of East Asian paternal lineages. Even in modern Hàn Chinese populations, however, the molecular marker associated with the spread of a Trans-Himalayan father tongue from the eastern Himalayan region, i.e. haplogroup O2a2b1 (M134) and its subclade O2a2b1a1 (M117), occurs in a much higher frequency than any other O haplogroup subclade, and approximately twice as frequently as the next most frequent fraternal subclade O2a1c (002611) (Yan *et al.* 2011; Wang *et al.* 2013; Yao *et al.* 2017b).

In autosomal terms, the Hàn ethnicity arose through incessant gene flow within successive dynastic empires, with their geographically ever shifting centres (Chiang *et al.* 2018). Hamada (2006) has shown how local and private concerns with regard to ethnic identity have been projected onto the past and thus distorted the interpretation of anthropological, archaeological and linguistic data in the Japanese context. The same may continue to be said today of laymen and even scholars projecting modern Hàn ethnic identity onto the past despite the admonitions of the eminent Chinese archaeologist Kwang-chih Chang (1986) to avoid the anachronisms that arise from applying the label ‘Chinese’ to archaeological cultural assemblages or peoples of the distant past.

The centre of Trans-Himalayan linguistic diversity lies in the eastern Himalayan region, more particularly on the southern flanks of the great Himalayan divide, where most of the languages of the family and over three-quarters of all currently recognised primary linguistic subgroups of the family are found. The aberrant nature of some Sinitic lexicons has long indicated to the minds of many an historical linguist that Sinitic must have arisen through creolisation when an ancient Trans-Himalayan speaking population first moved to the already populated North China plain (Poppe 1965; Benedict 1972; Hashimoto 1976a, 1976b, 1980, 1986; Ballard 1979; Norman 1982; Comrie 2008; DeLancey 2011). Genetic data have newly begun to lend support to this linguistic hypothesis (He *et al.* 2019).

Findings of ancient DNA studies in this regard form the topic of one of our forthcoming studies. Much later, at the far shallower time depth of the Qín dynasty in the third century BC, this ethnicity spread southward from the Yellow River basin into southern China (Wen *et al.* 2004), where this martial and male-biased historical spread during the cultural sinification of the region south of the Yangtze involved both the spread of language and the introduction of paternal lineages, as historically documented in the Chinese chronicles.

## The peopling of Japan

**Figure 5.**
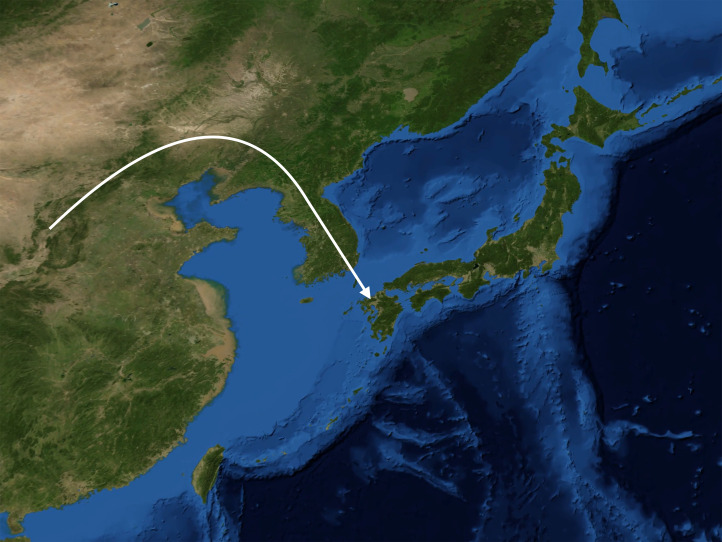
In successive waves, the paternal lineages D1a2 (M55), C1a1 (M8), C2 (M217) and O1b2 (M176) migrated from the East Asian mainland to the Japanese archipelago at the dawn of the Palaeolithic, the Incipient Jōmon, the Early Jōmon and the Yayoi period respectively.

Our original reconstruction of the peopling of Japan (van Driem 2014), based on earlier genetic research (Kivisild *et al.* 2002; Tajima *et al.* 2004; Tanaka *et al.* 2004; Hammer *et al.* 2006; Jin *et al.* 2009; Karafet *et al.* 2009), has been borne out – and can now also be fleshed out – by additional work on modern Japanese DNA (Mabuchi *et al.* 2007; Nonaka *et al.* 2007; Yamaguchi-Kabata *et al.* 2008; Nohira *et al.* 2008; Pen and Zhang 2012; Poznik *et al.* 2016) and on ancient Japanese and East Asian DNA (Shinoda 2004; Xue *et al.* 2006; Shinoda and Doi 2008; Adachi *et al.* 2009, 2011, 2013; Igawa *et al.* 2009; Kim *et al*. 2011; Jinam *et al.* 2012, 2015; Kanzawa-Kiriyama *et al.* 2013; Trejaut *et al.* 2014; Nakagome *et al.* 2015; Yao *et al.* 2017a, c; Saitou & Jinam 2017; Adachi *et al.* 2018; Watanabe *et al.* 2019; Gakuhari *et al.* 2019). The synoptic reconstruction presented here and in Figure 5 embodies a number of hypotheses, which may be corroborated or refuted by future ancient DNA findings, or perhaps just require minor reformulation.

Palaeolithic hunter–foragers bearing the paternal lineage D1a2 (M55) and speaking a language ancestral to Ainu settled Japan about 38,000 years ago, bringing with them the oldest Palaeolithic tools now found in the archipelago. This paternal lineage is retained throughout Japan and particularly survives in a high frequency on the Ryūkyū islands and in a very high frequency of over 80% amongst the Ainu of Hokkaidō, whom ethnographic accounts have described as hirsute and phenotypically distinct from other Japanese. Palaeolithic settlements on the main Japanese islands appear over 34,000 years ago (Mizoguchi 2002, 2014). In the Ryūkyū archipelago, palaeolithic settlements on the Amami and Okinawa island groups are likewise attested from 34,000 years ago. On the Sakishima islands, palaeolithic cultural assemblages in the Miyako and Yaeyama island groups are attested from 27,000 years ago. In the Amami and Okinawa island groups, archaeological strata reflecting the Early Shellmound period begin at about 9000 years ago, whereas in the southern portion of the Ryūkyū archipelago the archaeological strata identified as reflecting the enigmatic Shimotabaru period begin about 4900 years ago (Pearson 2013; Akamine 2017).

Philip von Siebold (1858) argued that the ancestors of the Ainu had originated in the Amur basin at a time that preceded the advent of Japonic speakers, whose subsequent arrival to the archipelago compelled the Ainu to migrate northward (von Siebold 1858: 380). The toponymical studies of Chamberlain (1887) and Batchelor (1925) showed that most old place names on Hokkaidō and numerous place names in northern and central Honshū were Ainu toponyms, with those ending in *-betsu* or *-be* [<Ainu *pet* ‘river’] and in *-nai* [<Ainu *nai* ‘stream’] being amongst the most conspicuous.

Subsequently, bearers of the paternal lineage C1a1 (M8) arrived in the archipelago and introduced the Incipient Jōmon culture, typified by early ceramic cultures such as the Ōdai Yamamoto i site. This paternal lineage is borne by 10% of modern Japanese men. At the dawn of the Early Jōmon period, bearers of the paternal lineage C2 (M217) arrived in Japan speaking the ancient Japonic language, which ultimately gave rise to modern Japanese and the Ryūkyūan dialects. This paternal lineage is borne by 6% of modern Japanese men.

Kæmpfer (1729: 63–65) identified the Amur river basin as the Altaic homeland, whence the linguistic ancestors of the Japanese had migrated to the archipelago via the Korean peninsula, an idea also espoused by latter-day linguists, e.g. van Driem (2001) and Robbeets (2014). The dual nature of Japanese population structure was advanced by Miller (1971), who proposed that the resident Jōmon population spoke an Altaic language ancestral to modern Japanese, and this Altaic tongue underwent Austronesian influence when the islanders absorbed the bearers of the incursive Yayoi culture. The Altaic linguistic phylum comprises Japonic, Korean, Tungusic, Mongolic and Turkic, but Robbeets (2014) reserves the label ‘Altaic’ for a putative clade which she believes comprises Tungusic, Mongolic and Turkic, and introduced the new label ‘Trans-Eurasian’ for the linguistic phylum traditionally called Altaic (Blažek *et al.* 2019).

About 3000 years ago, the bearers of the O1b2 (M176) paternal lineage came to Japan from the Korean peninsula, introducing rice cultivation and appearing archaeologically as the Yayoi. In their wake, bearers of other O haplogroup subclades prevalent on adjacent portions of the East Asian mainland also migrated to the archipelago. In time, the Yayoi sedentary agricultural lifestyle prevailed, and Yayoi paternal lineages came to predominate and today account for over half of all Japanese paternal lineages, with the highest frequencies in Kyūshū. Yet the gracile Yayoi newcomers with their farming subsistence strategy, notwithstanding their superior bronze and iron metallurgy, were evidently motivated or compelled to assimilate linguistically to the robust Japonic speakers already on the island.

The Japonic-speaking Early Jōmon people must have been drawn in to avail themselves of the pickings of Yayoi agricultural yields, and the Yayoi may have prospered and succeeded in multiplying their paternal lineages precisely because they managed to accommodate the Jōmon linguistically and in material ways. In addition to rice, the Yayoi introduced other crops of continental inspiration to the Japanese archipelago such as millet, wheat and melons. Their ancestors had certainly encountered these crops on their way northward to the Korean peninsula, the earliest attested domestic millet dating from before 6000 BC at Xīnglōnggōu near Chìfēng, where a Neolithic culture without sickles has been described (Zhào 2005).

The original ‘para-Austroasiatic’ tongue of the Yayoi was lost except perhaps for loan words denoting agricultural terms (cf. Benedict 1990). Notwithstanding a possible Austronesian presence in the Sakishima islands from Formosa at the end of the third millennium BC (Hudson 2017), any alleged ‘Austronesian’ influence on Japonic (Polivanov 1918, 1924; van Hinloopen Labberton 1924, 1925; Whymant, 1926; Benedict 1990) would have had to antedate the arrival of the Yayoi in Japan, deriving from the Lóngshān interaction sphere connecting the Dàwènkǒu culture of Shāndōng with Formosa and other coastal cultures, e.g. Qīngliángǎng in northern Jiāngsū, Mǎjiābāng in the Yangtze delta.

## The legacy of lost father tongues and the spread of agriculture

We previously took issue with the hypothesis that the founding dispersals of language families coincided with the spread of agriculture in a volume edited by Bellwood and Renfrew, two staunch proponents of this very hypothesis (van Driem 2002). Quite to the contrary, the spread of Indo-European furnishes the most obvious counter-example. Likewise, Sumerian, Elamite, Akkadian, Hurrian, Hattic and other languages which have left no surviving linguistic descendants were the tongues spoken by early agricultural civilisations, which therefore bear witness to the permeability of linguistic boundaries for the dissemination of agriculture.

The Neolithic and Bronze Age of Asia Minor and Mesopotamia are characterised by a very long period of incursive population movements into, rather than out of, Anatolia and the Fertile Crescent, driven or lured by the relative affluence of urban centres supported by agricultural surplus. Not just Indo-Europeans such as Hittites and Mitanni were drawn in by the good life. Gutaeans, Amorites, Kassites and other peoples likewise came to settle in the Fertile Crescent and Anatolia. Toponymical evidence and details about the cults of certain deities have been used to argue that even the Sumerians originally migrated from an earlier northern homeland to lower Mesopotamia, where they adopted agriculture from a resident population, at which time the Sumerians borrowed agricultural terms such as *agar* ‘field’, *apin* ‘seeder plough’ and *apsin* ‘furrow’ from a substrate language (Landsberger 1943, 1944, 1945, 1974; cf. Rubio 1999).

Similarly, it is likely that the Trans-Himalayans who introduced their pre-Sinitic language to the Yellow River basin first came as migrants in search of the good life to the affluent agriculture societies on the North China plain. It is therefore not inconceivable that the Yellow River basin, where *Setaria* and *Panicum* began to be domesticated about nine millennia ago, could have been the original Altaic homeland. This primary homeland may have been abandoned after an ancient Trans-Himalayan population introduced themselves and their Proto-Sinitic language to the early inhabitants of the North China plain, after which the secondary homeland of Trans-Eurasian may have moved north towards the Amur river basin.

The Father Tongue correlation can likewise not explain everything. If the paternal lineage C2 (M217) is correlated with Altaic linguistic affinity, as appears to be the case for Turkic, Mongolic and Tungusic, then Japanese is no Father Tongue, and neither is Korean. This Y-chromosomal haplogroup accounts for 11% of Korean paternal lineages, and the frequency of the lineage is even more reduced in Japan. Yet this molecular marker may still be a tracer for the introduction of Altaic language to the archipelago, where the paternal lineage has persisted, albeit in a frequency of just 6%.

On the other hand, the Y-chromosomal haplogroup D1a2 (M55) appears to be correlated with the ancient linguistic phylum of which Ainu is the surviving remnant. Therefore, Ainu is a father tongue, and the ancient paternal lineage D1a2 (M55) also remains robustly present elsewhere in Japan. If the bearers of the paternal lineage O2b introduced Yayoi culture and wet rice cultivation to the Japanese archipelago, then agriculture and superior metallurgy appear to have contributed to the fecundity of this paternal lineage, a veritable agricultural spread but without language.

Overly simple approaches that turn up correlations where direct causation is lacking or unlikely have come to enjoy perennial popularity (e.g. Nettle 1998; Gorenflo *et al.* 2012; Axelsen and Manrubia 2014; Greenhill *et al.* 2018; Hua *et al.* 2019). The Farmer Language Dispersal model is no doubt superior to such approaches in seeking to understand the dispersal of language families in terms of the spread of a subsistence strategy that has changed the face of our planet. However, enthused scholars oblivious to the faulty nature of the reasoning underlying the Farmer Language Dispersal model are inclined to seek corroboration and reinterpret evidence in ways that ‘fit’ that model. Similarly, the false assumption that any widely observed phenomenon, such as the Father Tongue correlation must therefore be universal, a presumption which we have repeatedly disavowed in print, would likewise prevent us from discerning the contours of a more complex picture of the past and render us unable to find the right fit for the plentiful pieces of the puzzle of prehistory.
